# Autapse-induced multiple coherence resonance in single neurons and neuronal networks

**DOI:** 10.1038/srep30914

**Published:** 2016-08-02

**Authors:** Ergin Yilmaz, Mahmut Ozer, Veli Baysal, Matjaž Perc

**Affiliations:** 1 Bülent Ecevit University, Department of Biomedical Engineering, Zonguldak, 67100, Turkey; 2 Bülent Ecevit University, Department of Electrical-Electronics Engineering, Zonguldak, 67100, Turkey; 3 Faculty of Natural Sciences and Mathematics, University of Maribor, Koroška cesta 160, SI-2000 Maribor, Slovenia

## Abstract

We study the effects of electrical and chemical autapse on the temporal coherence or firing regularity of single stochastic Hodgkin-Huxley neurons and scale-free neuronal networks. Also, we study the effects of chemical autapse on the occurrence of spatial synchronization in scale-free neuronal networks. Irrespective of the type of autapse, we observe autaptic time delay induced multiple coherence resonance for appropriately tuned autaptic conductance levels in single neurons. More precisely, we show that in the presence of an electrical autapse, there is an optimal intensity of channel noise inducing the multiple coherence resonance, whereas in the presence of chemical autapse the occurrence of multiple coherence resonance is less sensitive to the channel noise intensity. At the network level, we find autaptic time delay induced multiple coherence resonance and synchronization transitions, occurring at approximately the same delay lengths. We show that these two phenomena can arise only at a specific range of the coupling strength, and that they can be observed independently of the average degree of the network.

Autapse is an unfamiliar synapse, which happens between the axon and soma of the same neuron, and forms a time-delayed self-feedback mechanism. This special kind of synapses on the nervous system was firstly explored by Van der Loos and Glaser^1^, and they called it as *autapse*, meaning the self-synapse. Thereafter, the existence of autapses in different brain regions was notified in various experimental studies by using different experimental techniques^2^,^3^,^4^. By using biocytin filling method, which is used for determining the activity and shape of the neuron, Tamas *et al*.^5^ demonstrated that inhibitory interneurons in the visual cortex can form between 10–30 autaptic connections. Lübke *et al*.^6^ showed that the majority of cortical pyramidal neurons (more than 80%) in the developing neocortex of a human brain have autaptic connections. Using perforated-patch clamp technique in their experimental study Bacci *et al*.^7^ uncovered the existence of GABAergic autaptic activities of the fast-spiking interneurons in the neocortical slices of the layer V.

On the other hand, in addition to studies in which the existence of the autapses has been presented, there are some studies where the effects of autapses on the neuronal dynamics are analyzed. For example, Bacci and Huguenard^8^ experimentally showed that an inhibitory autaptic transmission has a considerable role in determining the spike-timing precision of the neocortical inhibitory interneurons. The spiking dynamics of a single neuron can be effectively modulated by an autapse modeled as an electrical or a chemical synapse^9^,^10^ Masoller *et al*.^11^ demonstrated the role of electrical self-connection, that is, electrical autapse, on the spiking dynamics of a thermoreceptor neuron in the presence of noise and subthreshold activity. Wang *et al*.^12^ showed that the autaptic feedback activity can switch the firing mode of Hindmarsh-Rose (HR) neuron among quiescent, periodic and chaotic firing states. Sainz-Trapaga *et al*.^13^ demonstrated that a self-feedback initiates spikes by enhancing the amplitude of the subthreshold oscillations above the threshold. Qin *et al*.^14^,^15^ showed that a negative feedback can lead to defects in the network by suppressing exciting neurons while a positive feedback is likely to promote excitations and induce mode transitions by generating continuous signal sources that ultimately lead to pulse or target waves, or even spiral waves under an appropriate noise intensity. In a recently published study, it was shown that the weak signal detection capacity of a single HH neuron can be significantly enhanced by an electrical self-feedback connection for proper values of its parameters^16^. Yilmaz *et al*.^17^,^18^ demonstrated that the propagation of pacemaker activity can dramatically be improved with aid of electrical autapse in small-world and scale-free neuronal networks when the imposed rhythms by the autapse, intrinsic dynamics and weak signal are locked.

Coherence resonance (CR) is an important finding emerging in many fields of science, including complex neuronal systems, where an intermediate level of noise amplifies the intrinsic oscillation signal of nonlinear systems^19^,^20^,^21^. Ion channel noise originating from random open-close fluctuations of ion channels is an important noise source in neurons. Thus, its effects on the neuronal dynamics have been investigated in detail. In particular, the emergence of multiple CR (MCR) phenomenon induced by information transmission delay and time periodic coupling strength in the presence of channel noise is reported in various theoretical works^22^,^23^,^24^. For instance, Gong *et al*.^25^ demonstrated that potassium channel blocking is more encouraging for the occurrence of delay-induced multiple CR than sodium channel blocking. Yilmaz *et al*.^23^ reported the occurrence of MCR phenomenon induced by the frequency of time-periodic coupling strength at the optimal noise intensity. Wang *et al*.^26^ analyzed the spatial coherence resonance (SCR) phenomenon and obtained maximally ordered spatial dynamics for short delay times at an intermediate range of additive noise.

Otherwise, synchronization is a common phenomenon emerging in many fields of science including complex neuronal networks. Due to the synchronous oscillations of neurons in the brain are thought to be associated with pathological brain functions including several neural diseases^27^,^28^ and underline many higher order brain functions, such as sensory integration, movement initiation and memory formation^29^ much efforts is devoted to the investigation of synchronization phenomena in neuronal networks, and reported many sources inducing synchronization and synchronization transitions in neuronal networks, such as information transmission delay induced synchronization^30^,^31^, synchronization transitions due to time delay, coupling strength and noise^32^,^33^,^34^,^35^.

Despite the above studies demonstrating the effects of autapses on the temporal response of a neuron, their functional significance in information processing and coding is not yet clearly known. Therefore, here our aim is to analyze the effects of autapse on the temporal coherence of single stochastic Hodgkin-Huxley (HH) neuron and scale-free neuronal networks, and to also reveal its effects on the spatial synchronization of HH neurons in scale-free neuronal networks. It is shown that the autaptic time delay can induce CR and MCR phenomena for proper values of the auatptic conductance, irrespective of the type of the autapse. Also, the autaptic time delay induced synchronization transitions are found in scale-free neuronal networks for small and intermediate coupling strengths, and these transitions are robust the variations in the network average degree. We present detailed results in the next section, while for details concerning the employed mathematical model and the analysis, we refer the reader to the Methods section further below.

## Results

### Effects of the electrical autapse on the temporal coherence of single neuron

**Figure 1:**
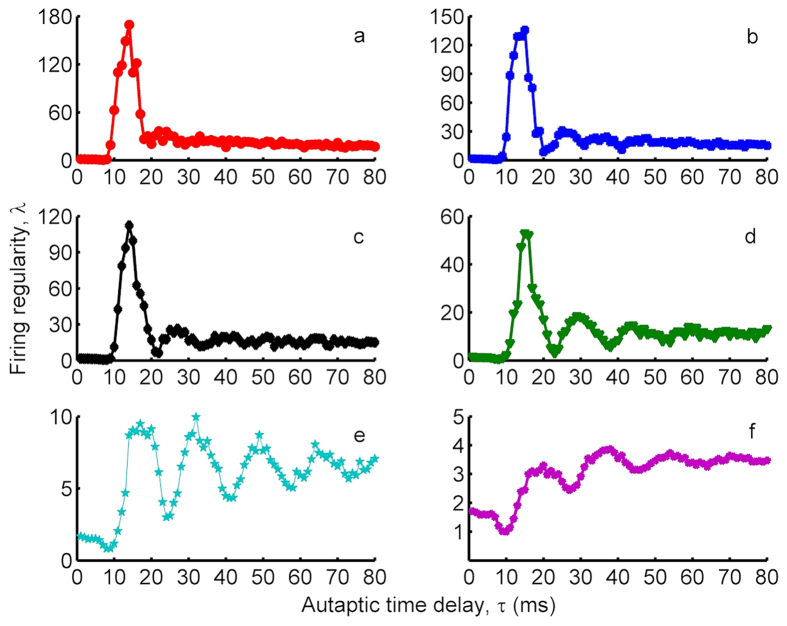
Dependence of the firing regularity *λ* on the autaptic time delay *τ* for different autaptic conductances *κ*. (**a**) *κ* = 0.7 *mS*/*cm*^2^, (**b**) *κ* = 0.6 *mS*/*cm*^2^, (**c**) *κ* = 0.5 *mS*/*cm*^2^, (**d**) *κ* = 0.4 *mS*/*cm*^2^, (**e**) *κ* = 0.3 *mS*/*cm*^2^, (**f**) *κ* = 0.2 *mS*/*cm*^2^ (*S* = 6 *μm*^2^). It is seen in the figure that, with increasing of autaptic conductance the best regularity increases, and the autaptic delay induced MCR phenomenon arises for the specific *κ* values. Also, each case of conductance level, the autaptic effect on the firing regularity saturates at longer delays and loses own effect.

In this subsection, how the electrical autapse affects the firing regularity or the temporal coherence of single stochastic HH neuron is investigated. For this purpose, we consider that the HH neuron has an autapse modeled as electrical autapse and add the autaptic current coming from this autaptic self-feedback connection to the neuronal dynamics by means of Eq. (13). We set the membrane patch size to 6 *μm*^2^. After that, we measure the firing regularity *λ* for various autaptic conductance values *κ*, depending on the autaptic time delay *τ* in Fig. 1.

Depending on *τ*, two different behaviors can observe on the firing regularity of the HH neuron as shown in Fig. 1. In the first one where *τ* < 12 *ms*, equaling roughly *refractory time interval* consumed during spiking and returning back the rest state, an autapse does not change or reduces the firing regularity below the one obtained without an autapse where spikes occur only due to the ion channel noise (in the absence of an autapse, the firing regularity is approximately equal to *λ* = 1.8 (data not shown)). Even, for specific *τ* values, it triggers a tendency to be escorted by a burst-like spiking on the output of HH neuron and this bursting causes very low *λ* values which have been reported in ref. 36. Also, in this sector, autapse hampers the occurrence of noise induced spiking by moving away the membrane potential from the threshold, and the total number of spikes thus falls below the one obtained without an autapse. In the second sector *τ* > 12 *ms*, the firing regularity exhibits coherence resonance (CR), double CR or multiple CR (MCR) phenomena depending on *τ* and *κ*. In strong autaptic conductance case (Fig. 1a), it shows the CR behavior with the highest resonance peak, whereas it exhibits a double CR and MCR with decreased *κ* (Fig. 1b–e) with smaller resonance peaks, depending on *τ*. But, the most prominent MCR behavior occurs relatively in small conductance case where *κ* = 0.3 *mS*/*cm*^2^. In addition, the resonance behavior of the firing regularity tends to disappear with increased autaptic time delay irrespective of the conductivity level of the autapse mechanism, and the regularity saturates at some values for relatively long autaptic delays.

**Figure 2:**
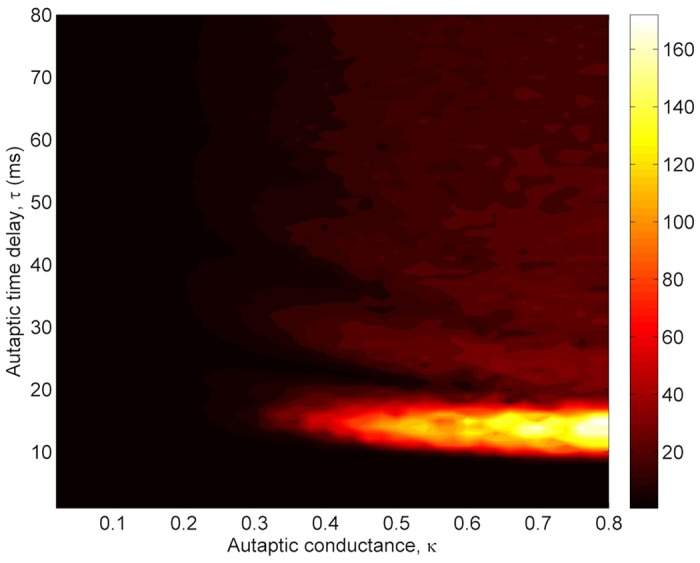
Contour plot of the firing regularity *λ* against the autaptic time delay *τ* and autaptic conductance *κ* in the presence of electrical autapse. We can easily see that there is a specific autaptic time delay interval, 10 *ms* < *τ* < 20 *ms*, in which the best regularity is obtained. Also, for the intermediate strengths of autaptic conductance the firing regularity passes some maximum and minimum with regard to increased *τ*, implying the occurrence of autaptic time delay induced MCR phenomenon. But, this MCR effect tends to disappear at long autaptic delays, and the autaptic activity can not induce MCR phenomenon.

To present a detailed view on the dependence of regularity on *τ* and *κ*, we show *λ* values at *τ*-*κ* parameter space in Fig. 2. The presented results in Fig. 2 verify the findings, which are obtained in Fig. 1. For *κ* values greater than *κ* ≥ 0.3 *mS*/*cm*^2^, there is a more pronounced resonance island emerging at roughly 10 *ms* < *τ* < 20 *ms*. This result implies that for specific values of *τ* and *κ*, an electrical autapse can significantly improves the firing regularity of a stochastic HH neuron. At the same time, we obtain MCR effect on the firing activity of the neuron for moderate levels of autaptic conductance.

**Figure 3:**
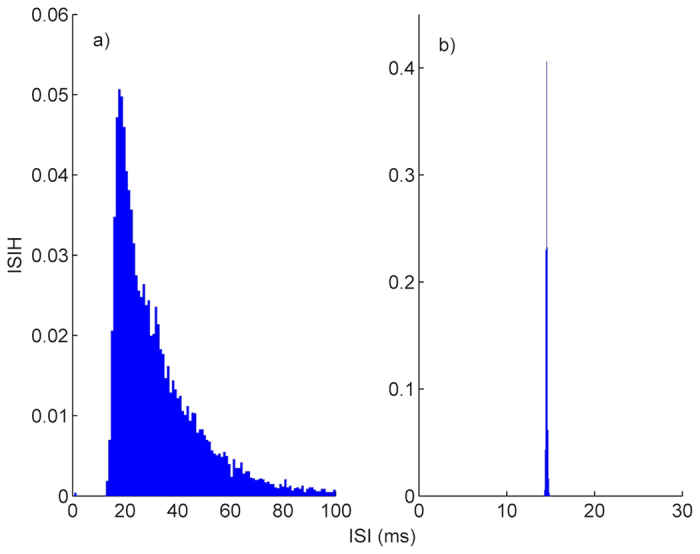
ISIH of single stochastic HH neuron. (**a**) in the absence of an autapse, (**b**) in the presence of an autapse with *κ* = 0.7 *mS*/*cm*^2^ and *τ* = 14 *ms*. In the absence of an autapse (only channel noise stimulates to neuron for spiking) ISIH is very broad with a peak located around the period (*T*_*osc*_ ≈ 21 *ms*) of subthreshold oscillations, but in the presence of an autapse the resulting ISIH is an unimodal and has a district peak around ISI of 14 *ms* which corresponds the *τ* at which the highest resonance peak is obtained (in Fig. 1). Readers should pay attention the axis scales of figures, which are given different for visibility.

To explain how an autapse increases the firing regularity of HH neuron, we give inter-spike interval histograms (ISIHs) computed from 10 000 ISIs in the presence of an autapse with *κ* = 0.7 *mS*/*cm*^2^ and *τ* = 14 *ms*, and in the absence of an autapse in Fig. 3. Figure 3a shows the ISIH of single stochastic HH neuron in the absence of an autapse, where spikes occur due to the channel noise. ISIH of single stochastic HH neuron is very broad, with a distinct peak near the subthreshold oscillation period (*T*_*osc*_ ≈ 21 *ms*)^37^ of HH neuron. In the presence of an autapse in Fig. 3b, the ISIH of the HH neuron exhibits a single, sharp peak centered around 14 ms which is equal to the autaptic time delay value at which the highest resonance peak is obtained (Fig. 1a). These results can be explained as follows: autapse adds a new time scale, which is not constant and varies depending on its parameters (i.e., *τ* and *κ*), to the neuronal dynamics. If this new time scale dominates the time scale introduced by the channel noise a new firing pattern emerges on the output of the neuron, where ISIH has a sharp peak implying the presence of dominant time scale in the spiking behavior of the neuron. Thereby, a high resonance peak is obtained in the firing regularity of the neuron.

### Effects of the chemical autapse on the temporal coherence of single neuron

**Figure 4:**
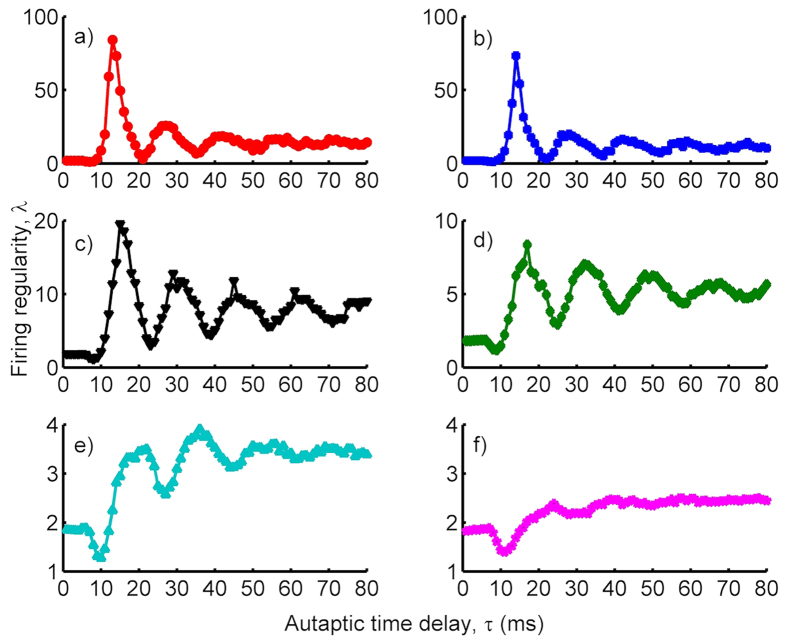
Dependence of the firing regularity *λ* on the autaptic time delay *τ* for different autaptic conductances *κ* in the presence of a chemical autapse (*S* = 6 *μm*^2^). (**a**) *κ* = 0.7 *mS*/*cm*^2^, (**b**) *κ* = 0.6 *mS*/*cm*^2^, (**c**) *κ* = 0.5 *mS*/*cm*^2^, (**d**) *κ* = 0.4 *mS*/*cm*^2^, (**e**) *κ* = 0.3 *mS*/*cm*^2^, (**f**) *κ* = 0.2 *mS*/*cm*^2^. The trend in *λ* is not changed prominently when the autapse is modeled as chemical synapse, but the best regularity is reduced as compared to the one obtained in case of electrical autapse. However, the MCR phenomenon is more likely to occur in case of the chemical autapse.

To investigate the effects of chemical autapse on the temporal coherence of single HH neuron, we consider that the HH neuron has an autapse modeled as chemical autapse. Self-feedback current originating from this autaptic connection is attached to the neuronal dynamics via Eqs (14 and 15). Obtained results indicated that the firing regularity of single stochastic HH neuron in the presence of a chemical autapse exhibits patterns similar to that of a neuron with an electrical autapse as seen in Fig. 4, where the resonance peaks have smaller amplitudes as compared to the neuron with an electrical autapse, and MCR behaviors are more prominent. At small autaptic conductance *κ* = 0.2 *mS*/*cm*^2^ (Fig. 4f), *λ* does not change prominently and stands nearly constant against increased *τ*. But, with the increase of *κ, λ* exhibits an oscillation-like behavior, where the amplitudes of oscillations attenuate against increasing *τ*, and *λ* saturates some values bigger than that of the neuron without an autapse. From these results presented in Figs 1 and 4, we conclude that the presence of an autapse, regardless of whether it is a chemical or an electrical autapse, increases the temporal coherence or firing regularity of the stochastic HH neuron under the condition that *τ* > 12 *ms*.

**Figure 5:**
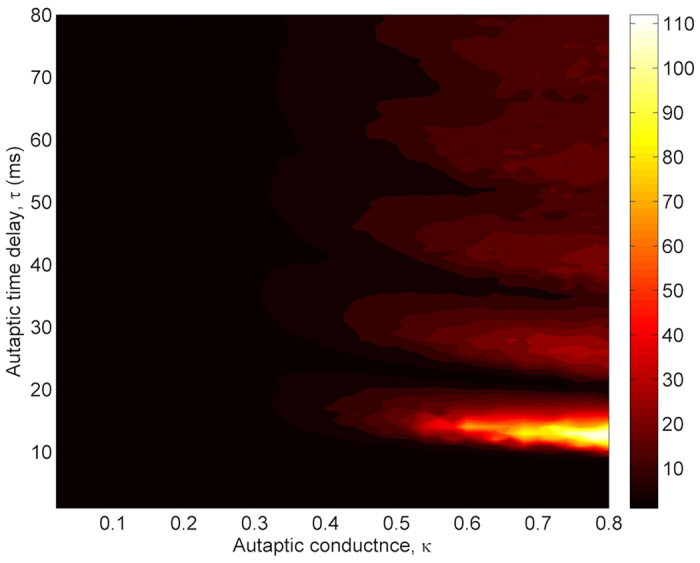
Contour plot of the firing regularity *λ* against the autaptic time delay *τ* and autaptic conductance *κ* in the presence of chemical autapse. While for some autaptic conductance levels, *κ* ≥ 0.3 *mS*/*cm*^2^, the autaptic activity can induce MCR phenomenon depending on autaptic time delay, it does not have prominent effects on the firing activity of the neuron for *κ* < 0.3 *mS*/*cm*^2^.

The dependence of *λ* on *τ* has been also studied for different *κ* values in Fig. 5, where a contour plot of *λ* at *τ*-*κ* plane is demonstrated. As *τ* increased, *λ* passes different resonance islands, indicating the high temporal coherence of the neuron, for *κ* ≥ 0.4 *mS*/*cm*^2^. This global view on (*τ*-*κ*)-plane verifies the obtained autaptic time delay induced MCR phenomenon in Fig. 4. When compared with the results obtained in the presence of an electrical autapse, both the resonance islands and resonance transitions are more distinct. However, although the highest resonance peak is obtained at approximately the same autaptic time delay value, the highest resonance amplitude is smaller in the presence of a chemical autapse. From this result, we make inferences that the electrical autapse has higher modulation capability on the spike discharge activity of the neuron as compared with the chemical autapse.

**Figure 6:**
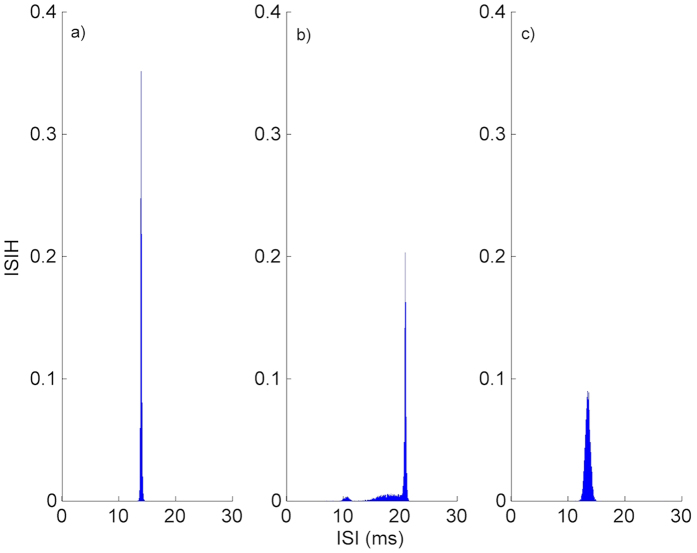
ISIH of single stochastic HH neuron in the presence of chemical autapse for *κ* = 0.7 *mS*/*cm*^2^. (**a**) with *τ* = 13 *ms*, (**b**) *τ* = 20 *ms*, (**c**) *τ* = 26 *ms*. Intrinsically, ISIHs occur at approximately the same ISI when *τ* is equal to the autaptic delay times at which the first and second resonance peaks emerge. As seen in figure, there is no matching between the period of intrinsic oscillations and the imposed time scale by autapse when the resonance peaks are obtained. Therefore, resonance phenomenon is a direct consequence of the autaptic effect dominating the neuronal dynamics.

To present the effects of the chemical autapse on the firing dynamics of a neuron, we show ISIHs of the neuron in Fig. 6a for *κ* = 0.7 *mS*/*cm*^2^ with *τ* = 13 *ms* representing the highest resonance peak in Fig. 4a, and with *τ* = 20 *ms*, and with *τ* = 26 *ms* which corresponds to second resonance peak in Fig. 4a. When *κ* = 0.7 *mS*/*cm*^2^ and *τ* = 13 *ms* (Fig. 6a), ISIH has a distinct, sharp peak with a highest peak value which emphasize the presence of a dominant time scale added by the autapse, referring to the more regular firings on the output of a neuron. The ISIs of a neuron with *κ* = 0.7 *mS*/*cm*^2^ and *τ* = 20 *ms* (Fig. 6b) are dispersed in a wider area embracing 10 *ms* < *ISI* < 22 *ms*, which indicates different time scales on the spike trains of the neuron, and the resulting regularity deteriorates. In Fig. 6c where *τ* = 26 *ms*, the ISIs occurs almost the same location as compared to Fig. 6a, but they distributed relatively a broader area with a smaller peak, which results in a more ordered spiking pattern compared with the findings obtained in Fig. 6b whereas it results in more irregular firings in a spike train as compared to Fig. 6a. These results state that autapse can be used as a control mechanism in adjusting the firing patterns of a neuron by introducing different time scales to the neuronal dynamics.

### Effects of the channel noise on the temporal coherence of single neuron

**Figure 7:**
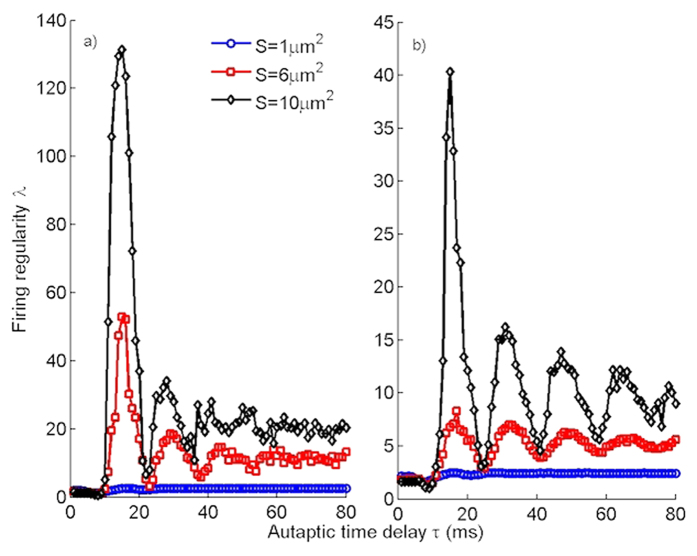
The firing regularity of a neuron for various channel noise intensities (*k* = 0.4 *mS*/*cm*^2^). (**a**) with an electrical autapse, (**b**) with a chemical autapse. At strong channel noise (*S* = 1 *μm*^2^), there is no prominent effect of the *τ* on *λ*, but with the decreasing of channel noise damped oscillations indicating the MCR phenomenon emerge, and further increase in *S* while the MCR effect disappears at long delay time in electrical autapse case, it continues to be observable in chemical autapse case.

Up to now, the effects of an autapse on the temporal coherence of single HH neuron have been investigated at fixed channel noise (*S* = 6 *μm*^2^). To illustrate how the channel noise affects the temporal coherence of HH neuron, the dependence of *λ* on *τ* is presented for various *S*, which scales the strength of channel noise, in Fig. 7a for the neuron with an electrical autapse and in Fig. 7b for the neuron with a chemical autapse. At strong channel noise (that is, *S* = 1 *μm*^2^), there is no prominent effect of the autapse on the temporal coherence of a neuron at both cases. This is due to that for *S* = 1 *μm*^2^ the channel noise is very strong and suppresses the time scale which is dictated to neuron by the autapse, and an irregular firings thus emerge on the output of a neuron. With the increase of *S* (*S* = 6 *μm*^2^), the strength of channel noise weakens and effects of the autapse on the firing dynamics begins to dominate, and consequently the temporal coherence of the neuron increases and autapse-induced MCR phenomenon emerges. At a relatively weak noise case (*S* = 10 *μm*^2^), the autapse becomes thoroughly dominant on the dynamics and high resonance peaks are obtained on the firing regularity of the neuron. Importantly, though similar trends emerges on the temporal coherence of the neuron with increased *S*, obtained *λ* values are greater in the presence of an electrical autapse as compared to that of in the presence of chemical autapse. Also, resonance peaks disappear for longer autaptic delay in case of electrical autapse and MCR behavior vanishes. In the light of these results, it is concluded that although an electrical autapse is more efficient than a chemical counterpart on the improvement of temporal coherence of the stochastic HH neuron, it is not so promoter the occurrence of MCR behavior as compared to the chemical autapse.

### Scale-free neuronal networks

After examining the effects of both electrical and chemical autapse on the temporal coherence of a single neuron, we now investigate the effects of chemical ones at the network level by employing a scale-free topological interaction among neurons. For this aim, we assume that each neuron in the network has one autapse modeled as chemical synapse, which is mathematically defined by Eqs (14 and 15), and add these autaptic currents to each neuron’s membrane potential via 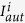 term in Eq. (17). We fix the membrane patch size to 6 *μm*^2^ as in single neuron case. Then, firstly we analyze the effects of autapse parameters on the temporal coherence of scale-free neuronal networks. After that we systematically investigate the effects of variations in coupling strength (*ε*) and in average degree of the network (*k*_*avg*_) on the temporal coherence of the network depending on autaptic time delay, *τ*. We also study how the spatial synchronization *σ* among neurons is affected by these parameters in the presence of autapses.

#### Effects of autaptic conductance and autaptic time delay on the temporal coherence and spatial synchronization of scale-free neuronal networks

**Figure 8:**
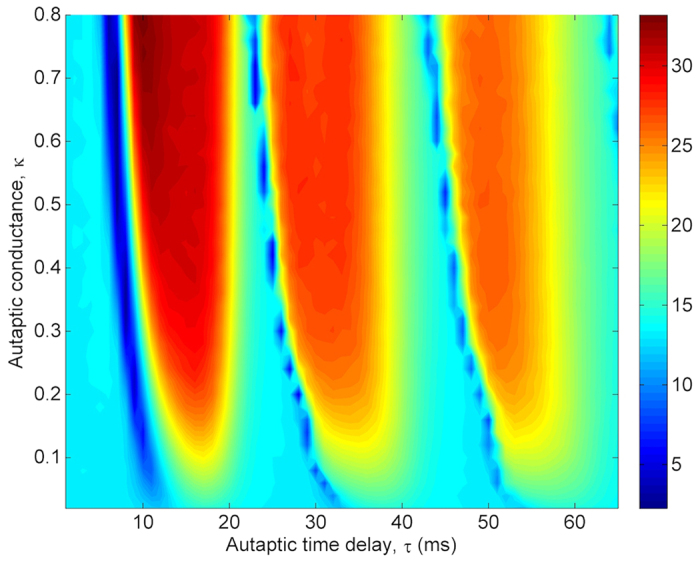
Dependence of the firing regularity *λ* on the autapse parameters, i.e., the autaptic time delay *τ* and autaptic conductance *κ*, in a scale-free network (*S* = 6 *μm*^2^, *k*_*avg*_ = 10, *ε* = 0.1). As can be observed, with the increasing autaptic time delay the firing regularity first diminishes, then the autaptic activity can intermittently induce resonance for specific ranges of the autaptic time delay, implying the autaptic time delay induced MCR phenomenon. Strikingly, when the *τ* approximately equals to *T*_*osc*_ or its integer multiples, autapse either does not improve the regularity or reduces it below the one obtained without autapse. Also the amplitude of resonance peaks decrease with increased autaptic time delay.

**Figure 9:**
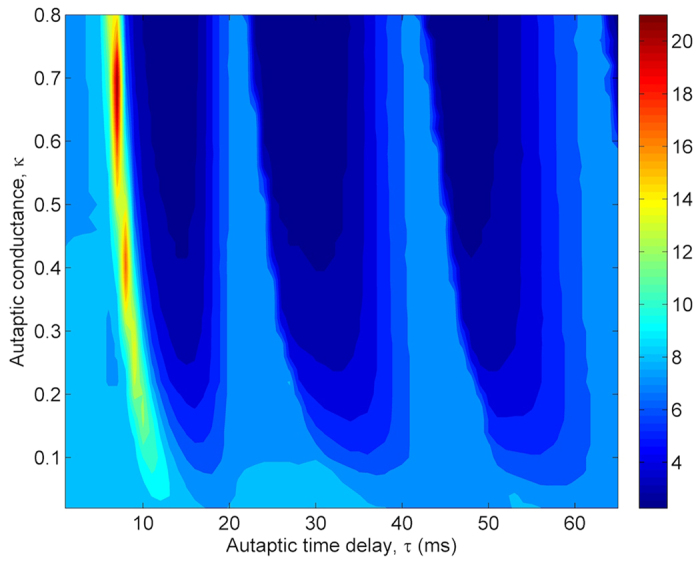
Contour plot of the spatial synchronization *σ* against the autaptic time delay *τ* and autaptic conductance *κ* in a scale-free network (*S* = 6 *μm*^2^, *k*_*avg*_ = 10, *ε* = 0.1). Autaptic activity firstly reduces synchronization for specific autaptic time delays, then it can lead to the occurrence of synchronization transitions with the increasing of autaptic time delay. Interestingly, for autaptic time delays equaling roughly the intrinsic oscillation period of the neuron or its integer multiples synchronization among neurons deteriorates. Besides, synchronization transitions is more evident at greater autaptic conductance levels.

Primarily, to demonstrate how the temporal coherence and spatial synchronization of a scale-free network change with the autapse, the alteration of *λ* and *σ* at *τ*-*κ* plane are presented in Figs 8 and 9, respectively. As seen in Fig. 8, as the autaptic time delay *τ* is increased, the temporal coherence or the firing regularity of the network passes through different resonance peaks (around *τ* ≈ 15 *ms*, 30 *ms*, 50 *ms*) represented with red shaded regions and non-resonance valleys (around *τ* ≈ 22 *ms*, 42 *ms*, 62 *ms*) indicated by blue shaded areas for *κ* > 0.1, indicating occurrence of the autaptic time delay induced MCR behavior in scale-free networks. Importantly, with increased *τ*, firstly, the autapse decreases the firing regularity of the network below one where neurons do not have autapse in the network (in this case, *λ* ≈ 12 in Fig. 2 of ref. 38), due to the tendency of burst-like spiking of individual neurons and decreasing synchronization among neurons as shown in Fig. 9. This behavior emerges smaller *τ* values as *κ* increases in the first valley, and intermittently occurs at specific *κ* values in the second and third valley. However, an opposite behavior is exhibited by the neuron against the increased values of the autapse parameters. With the increasing of autaptic time delay the resonance amplitudes decrease whereas with the increasing of auatptic conductance they increases.

In Fig. 9, where the dependence of spatial synchronization *σ* on *κ* and *τ* is analyzed. *σ* shows similar trends as in *λ*. At first, it increases against increased *τ*. Then it sharply decreases and passes various minimum (around *τ* ≈ 15 *ms*, 30 *ms*, 50 *ms*) and maximum (around *τ* ≈ 22 *ms*, 42 *ms*, 62 *ms*) implying the synchronous and non-synchronous state of the neurons in the network, respectively, and emphasizing the synchronization transition induced by autaptic time delay, *τ*. These results are consistent with those of presented in ref. 39. Interestingly, when the autaptic time delay is nearly equal to the intrinsic oscillation period (*T*_*osc*_) of the stochastic HH neuron or its integer multiples, *σ* increases or the synchronization among neurons decreases, and consequently the temporal coherence of the network reduces. This is due to the difference between rhythms imposed by the channel noise and autapse.

On the other hand, some recent works have reported the occurrence of autaptic time delay induced multiple stochastic resonance (MSR) phenomenon in single neuron^16^ and in neuronal networks with pacemaker^17^,^18^, when the time scales dictated by autapse, intrinsic dynamics (*T*_*osc*_) and the period of weak signal are matched. But, in the current study, the reason behind emerging of CR and MCR is different from that of MSR. In the former, there is a matching among time scales dictated to the neuron, whereas in the latter there is no matching between time scales on the resonance islands.

#### Effects of coupling strength on the temporal coherence and spatial synchronization of scale-free neuronal networks

**Figure 10:**
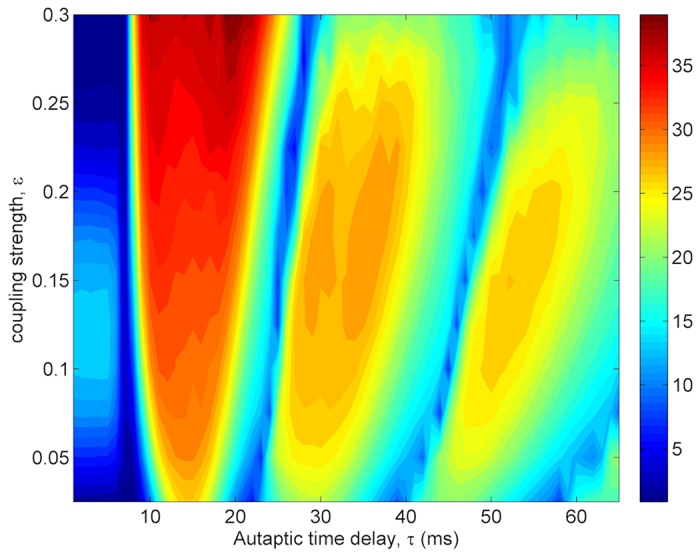
Contour plot of the regularity *λ* against the autaptic time delay *τ* and coupling strength *ε* in a scale-free network (*S* = 6 *μm*^2^, *k*_*avg*_ = 10, *κ* = 0.5 *mS*/*cm*^2^). We obtain different resonance islands represented with red shaded areas, in which autapse triggers more ordered spiking patterns on the out of neuron, depending on autaptic time delay. Interestingly, when the autaptic time delay roughly equals to the intrinsic oscillation period of the neuron or its integer multiples the firing regularity decreases to the values being smaller than without autapse. Besides, for strong and weak coupling strengths the second and third resonance islands start to disappear. We infer that autaptic time delay induced MCR effect is not a robust phenomenon against the fluctuations in the coupling strength.

**Figure 11:**
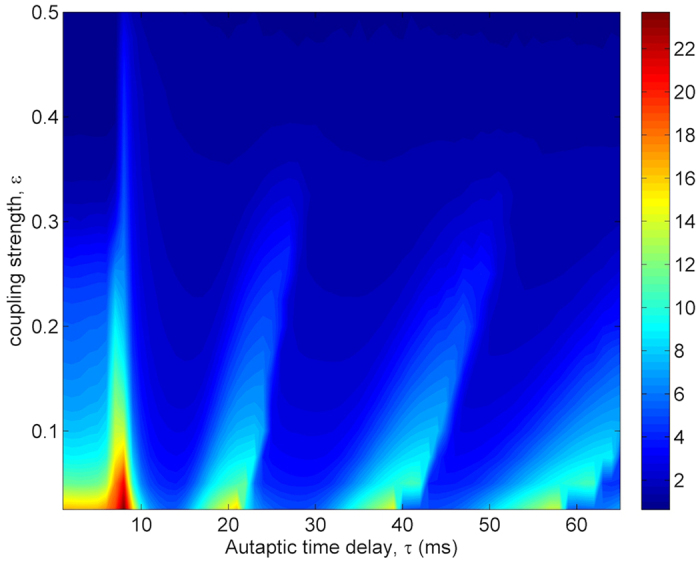
Contour plot of the spatial synchronization *σ* against the autaptic time delay *τ* and coupling strength *ε* in a scale-free network (*S* = 6 *μm*^2^, *k*_*avg*_ = 10, *κ* = 0.5 *mS*/*cm*^2^). We can see that in intermediate and weak coupled networks auatptic activity can induce synchronization transitions depending on autaptic time delay. But, at strongly coupled networks, there is no synchronization transitions. This is because increased *ε*, that is, increased coupling current establishes strong spatial synchronization among neurons, and the autaptic current loses own influence on the dynamics and can not break synchronization. In this circumstances, the only force that can act on the firing behavior of neuron is the coupling current coming from the other neurons in the network. As a result of this conditions synchronization transitions can not be induced.

To demonstrate the effects of coupling strength *ε* on the temporal coherence of the network and on the synchronization in the presence of autapses, the dependence of *λ* and *σ* on *ε* and *τ* is depicted in Figs 10 and 11, respectively. For this analysis, we choose the autaptic conductance as *κ* = 0.5 *mS*/*cm*^2^ and the network average degree as *k*_*avg*_ = 10. As seen in Fig. 10, with the increasing of *ε* the amplitudes of resonance peaks increase gradually for small and intermediate coupling strength and the MCR behavior is enhanced, but with the further increase in *ε* the second and third resonance islands start to disappear, consequently the autaptic delay induced MCR behavior fades away and only CR effect remains. This result show that there is a range of *ε* which roughly covers 0.05 < *ε* < 0.25 for emerging of autaptic time delay induced MCR effect in scale-free neuronal networks.

When the dependence of *σ* on *ε* and *τ* is examined in Fig. 11, while the synchronization transitions induced by *τ* is more prominent for small *ε* (*ε* < 0.1), its evidence reduces for intermediate *ε* (0.1 < *ε* < 0.3) and begins to disappear for *ε* > 0.3. This is because increased *ε* enhances synchronization among neurons and constitutes a strong synchronization among neurons in the network, and the autaptic effect does not break this synchronization. Therefore, in a tightly coupled network, the autaptic time delay induced MCR phenomenon is unobservable.

#### Effects of network average degree on the temporal coherence and spatial synchronization of scale-free networks

**Figure 12:**
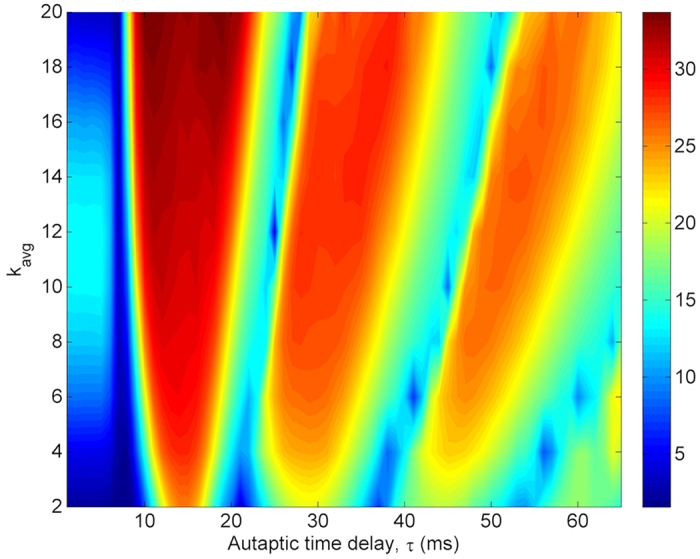
Contour plot of the regularity *λ* against the autaptic time delay *τ* and network average degree *k*_*avg*_ in a scale-free network (*S* = 6 *μm*^2^, *k*_*avg*_ = 10, *κ* = 0.5 *mS*/*cm*^2^). The MCR phenomenon is not much affected by the connection density of the network, and emerges independently from the network average degree. Only in densely connected networks the second and third resonance islands occur at larger delays with a little bit smaller peaks.

In Fig. 12, the dependence of the regularity on *τ* and *k*_*avg*_ is presented. It is seen that the collective activity of the network passes different resonance islands depicted the red shaded areas irrespective of the value of *k*_*avg*_, with regard to the increased auatptic time delay. Such a finding clearly represents the occurrence of autaptic time delay induced multiple coherence resonance in scale-free neuronal networks. In addition, while the second and third resonance islands emerges at long delays against increased *k*_*avg*_, the resonance amplitude does not improve except for the first resonance island. These results imply that autaptic time delay induced MCR behavior is a robust phenomenon, not to be affected by the connectedness degree of the network.

**Figure 13:**
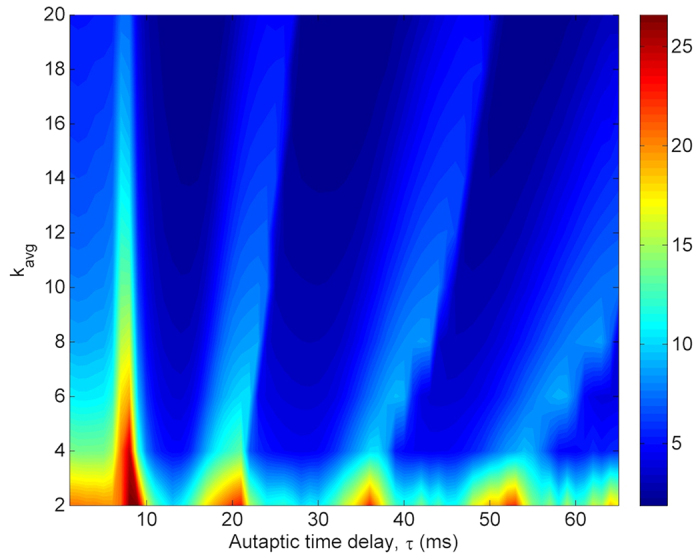
Contour plot of the spatial synchronization *σ* against the autaptic time delay *τ* and network average degree *k*_*avg*_ in a scale-free network (*S* = 6 *μm*^2^, *k*_*avg*_ = 10, *κ* = 0.5 *mS*/*cm*^2^). Synchronization transitions can be observable in the whole considered range of network average degree. But, in sparsely connected networks these transitions are more prominent whereas they are seen with less clarity in densely connected networks due to the established synchronization by the increased average degree.

In Fig. 13, the dependence of *σ* on *τ* and *k*_*avg*_ is shown. In relatively sparsely connected networks (*k*_*avg*_ < 10), the synchronization transitions are more sensible, and more robust against increasing *τ*. But, in densely connected networks (*k*_*avg*_ > 16), although the effect of autaptic activity triggering the synchronization transitions starts to lose its effectiveness due to increased synchronization among neurons, the synchronization transitions can still observe. When the results presented in Fig. 11 and in Fig. 13 are compared, both increasing coupling strength and increasing average degree display the same effect and enhance the extent of synchronization. However, the established synchronization by coupling strength is more persistent as compared to that of by average degree. Because, the synchronization transitions induced by autaptic activity do not arise in tightly connected networks, but they can be constituted in densely counterparts.

## Discussion

In sum, firstly, the effects of both chemical and electrical autapse on the temporal coherence or the firing regularity of a single stochastic HH neuron has been investigated. Then, the effects of chemical autapse on the temporal coherence and spatial synchronization of the neurons are examined in scale-free neuronal networks by assuming that each neuron in the network has one chemical autapse. At single neuron level, at both cases, viz. in the presence of electrical autapse and chemical autapse, the autaptic delay induced CR and MCR phenomena arise at fine tuned autaptic conductance levels. Further, with the increasing of *κ*, while the CR phenomenon enhances and remains approximately stable within the same *τ* range at both autapse cases, the MCR phenomenon disappears in the presence of an electrical autapse. We also investigate the effects of channel noise on the firing regularity of the HH neuron in the presence of both electrical and chemical autpase. The neuron with an electrical autapse exhibits enhanced CR effect with the decreasing of channel noise and needs an intermediate noise intensities to show MCR effect. Otherwise, the one with a chemical autapse displays enhanced MCR effect against decreasing channel noise intensity. At the network-level, for fixed coupling strength, *ε*, the autaptic time delay induced MCR is obtained within a broader range of autaptic conductance and is a more robust phenomenon against variations in autaptic conductance. Also, autaptic time delay induced synchronization transitions is obtained at almost same delay lengths. While the synchronization transitions are more prominent at larger autaptic conductances, they lose visibility at relatively small autaptic conductances. Besides, the impacts of coupling strength on the firing regularity and spatial synchronization have been analyzed and find that the CR effect improves against increasing coupling strength whereas the MCR effect gets lost at higher coupling strength. At the same time, synchronization transitions also disappear due to increased synchronization at strong coupling levels. Finally, we obtain that the CR and MCR phenomena are robust ones with respect to variations in average degree of the network, and the synchronization transitions are observable with less clarity against increasing average degree of the network.

Some part of our findings are consistent with the results presented previous experimental works. For example, fast spiking (FS) interneurons exhibit quite precise spike timing under favour of powerful GABAergic autaptic connections. It has been experimentally shown that blockade of this autaptic transmission reduces the temporal precision of spike timing in FS interneurons^8^. In the same study, it was shown that firing precision in layer V pyramidal neurons (normally they do not possess GABAergic autaptic connections and exhibit a low-spike timing precision) has been increased by adding artificial GABAergic autapse^8^. These findings provide a direct support to our results. Because, in the absence of an autapse, we obtain very broad ISIH implying a low-spike timing precision, but in the presence of an autapse with the proper parameters’ value we find a very sharp, distinct peak in ISIH of the neuron referring a high-spike timing precision.

In the current study, we consider that each neurons in the network has only an autapse which is modeled as a chemical synapse. But, in realistic circumstances, one neuron may have more autaptic connections^5^, which could be both electrical and chemical autapse. Therefore, in future studies, it is of interest to investigate the effects of hybrid autapses on the fascinating nonlinear phenomena emerging in neuronal networks. We hope that our findings will enhance our understanding of the effects of autapses on the neuronal dynamics.

## Methods

To capture neuronal dynamics in the presence of an autpase, the Hodgkin-Huxley neuron model, being biophysically detailed one, is used. Naturally, map-based models could be used as well for a different modeling perspective^40^, and similar methodology could also be used to study a different set of phenomena^41^. Here, the time evolution of the membrane potential of a neuron is given as follows:


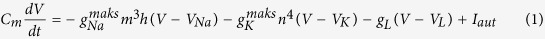


In Eq. (1), *C*_*m*_ = 1 *μF*/*cm*^2^ represents the capacity of cell membrane. *V* is the membrane potential of the neuron. 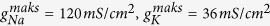 ve 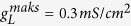 receptively denote the maximal sodium, potassium and leakage conductance when all ion channels are open. *V*_*Na*_ = 50 *mV, V*_*K*_ = −77 *mV* and *V*_*L*_ = −54.4 *mV* are the reversal potentials for the sodium, potassium and leakage current, respectively. The gating variables *m, n* and *h* in Eq. (1) represent the mean ratios of the open gates of the specific ion channels. The *n*^4^ and *m*^3^*h* are the mean portions of the open potassium and sodium ion channels within a membrane patch, and their dynamics are controlled by voltage-dependent opening and closing rates *α*_*x*_(*V*) ve *β*_*x*_(*V*) (*x* = *m, n, h*). The dynamics of gating variables depending on rate functions are given:


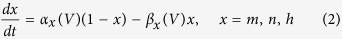



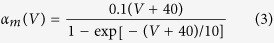



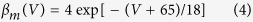



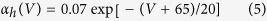



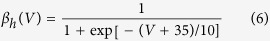



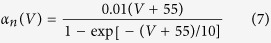



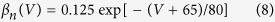


The above equations do not include ion channel noise originating from random open-close fluctuations of ion channels and are valid only at large membrane sizes. To include the effects of ion channel noise on the neuronal dynamic Langevin generalization for the dynamics of gating variables is used as below^42^


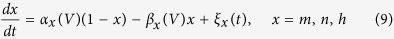


here, *ξ*_*x*_(*t*) is independent zero mean Gaussian white noise sources whose autocorrelation functions are given as below^42^


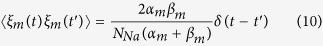



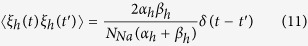



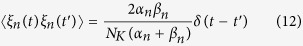


where, *N*_*Na*_ and *N*_*K*_ represent the total number of sodium and potassium channels in a given membrane size, and are calculated as *N*_*Na*_ = *ρ*_*Na*_*S, N*_*K*_ = *ρ*_*K*_*S* where *ρ*_*Na*_ = 60 *μm*^−2^ ve *ρ*_*K*_ = 18 *μm*^−2^ are the sodium and potassium channel densities, respectively. *S* represents the total cell membrane area. It is easily seen that channel noise is inversely proportional to cell membrane area *S*. In Eq. (1), *I*_*aut*_ denotes the autaptic self-feedback current. In the current study, both electrical and chemical autapse is considered for the kind of autapse. In electrical autapse case, the autaptic current is modeled by below equation^43^:


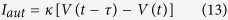


where *κ* denotes the autaptic conductance, and *τ* represents the autaptic time delay, which occurs because of the finite propagation speed during axonal transmission.

In chemical autapse case, the autaptic current is modeled using the so-called fast threshold modulation given by the following function^44^,^45^:


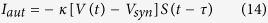



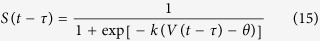


where, *V*_*syn*_ = 2 *mV* for excitatory chemical autapse, *k* = 8 and *θ* = −0.25.

To quantify the firing regularity or the temporal coherence of the spiking activity of a neuron, we use the inverse of coefficient of variation (CV), represented by *λ*, of the inter-spike intervals (ISIs):


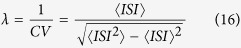


where 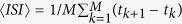 and 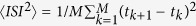 denote the mean and mean-squared ISIs, respectively. The parameter *t*_*k*_ is the time of *k*th spike in the spike train. A spike event is defined by the upward crossing of the membrane potential past a detection threshold of 20 *mV*.

For network level investigations, following the procedure used in ref. 46, we form the scale-free neuronal network (scale-free networks have been observed frequently in the realm of neuroscience, from functional networks to the connectome^47^,^48^) consisting of *N* = 200 neurons. In scale-free neuronal networks, the membrane potential of a neuron in the presence of an autapse is given with the following equation.


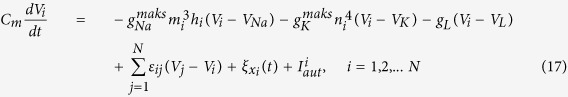



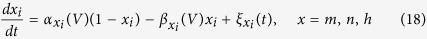


where *V*_*i*_(*t*) and *V*_*j*_(*t*) are the membrane potentials for neuron *i* and neuron *j*, respectively. 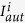 is the autaptic self-feedback current of the neuron *i*, which arise from the considered chemical autapse. *ε*_*ij*_ is the coupling strength between neuron *i* and neuron *j*. If neuron *i* and *j* are connected, *ε*_*ij*_ = *ε*, otherwise *ε*_*ij*_ = 0.

To measure the temporal coherence or the firing regularity of the spiking neuron in the network, inverse of the coefficient of variation of the inter-spike intervals of each neuron is used.


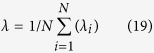



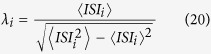


where *λ*_*i*_ measures the firing regularity of neuron *i* in the network (*i* = 1, 2,...*N*). 〈*ISI*_*i*_〉 and 〈*ISI*_*i*_〉^2^ denote the mean and mean-squared ISIs of the neuron *i*. Different from single neuron, in the network, the spiking threshold for a spike event is chosen as 0 *mv*. Higher *λ* corresponds to more ordered spiking pattern or higher firing regularity.

To measure the degree of spatial synchronization among neurons in the networks, the standard deviation *σ* is used and calculated as follow^32^:


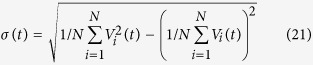



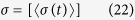


here 〈.〉 denotes the time averaging and [.] represents the averaging process over different network realizations. The smaller the *σ* the higher the synchronization in the network.

To ensure statistical consistency, all *λ* and *σ* values presented in the figures are calculated by averaging over 20 different realizations, each of which is 100 seconds, in single neuron and neuronal networks for the given parameter sets. All numerical integrals are solved using forward Euler method with the time step of 10 *μ*s. For initial conditions, we consider periodic boundary condition except the noise term.

## Additional Information

**How to cite this article**: Yilmaz, E. *et al*. Autapse-induced multiple coherence resonance in single neurons and neuronal networks. *Sci. Rep.* **6**, 30914; doi: 10.1038/srep30914 (2016).
